# Effect of the over-dominant expression of proteins on nicotine heterosis via proteomic analysis

**DOI:** 10.1038/s41598-021-00614-x

**Published:** 2021-10-26

**Authors:** Zejun Mo, Yuanyuan Pu, Junhao Zhou, Zonglin Tian, Jianhui Teng, Qian Chen, Lili Duan, Renxiang Liu

**Affiliations:** 1 College of Agriculture, Guizhou University, Guiyang, China; 2 College of Tobacco, Guizhou University, Guiyang, China; 3 Key Laboratory of Tobacco Quality in Guizhou Province, Guiyang, China

**Keywords:** Biological techniques, Genetics, Plant sciences

## Abstract

Heterosis is a common biological phenomenon that can be used to optimize yield and quality of crops. Using heterosis breeding, hybrids with suitable nicotine content have been applied to tobacco leaf production. However, the molecular mechanism of the formation of nicotine heterosis has never been explained from the perspective of protein. The DIA proteomics technique was used to compare the differential proteomics of the hybrid Va116 × Basma, showing strong heterosis in nicotine content from its parent lines Va116 and Basma. Proteomics analysis indicated that 65.2% of DEPs showed over-dominant expression patterns, and these DEPs included *QS*, *BBL*, *GS*, *ARAF* and *RFC1* which related to nicotine synthesis. In addition, some DEPs (including *GST*, *ABCE2* and *ABCF1* and *SLY1*) that may be associated with nicotinic transport exhibited significant heterosis over the parental lines. These findings demonstrated that the efficiency of the synthesis and transport of nicotine in hybrids was significantly higher than that in the parent lines, and the accumulation of over-dominant expression proteins may be the cause of heterosis of nicotinic content in hybrids.

## Introduction

The phenomenon of heterosis is accompanied by sexual reproduction, which means that some traits such as growth and development, reproduction rate, stress resistance, yield traits, and quality factors of the hybrid offspring are better than those of their parents^1^. As early as the early nineteenth century, researchers have proposed three genetic hypotheses of heterosis: dominance^2^, over-dominance^3^, and epistasis^4, 5^. Heterosis can give rise to new varieties and provide genetic benefits and has been widely used in corn, rice, wheat, and other crops^6, 7^. However, the research on the genetic basis of heterosis lags far behind the practical application, and the molecular mechanism of formation of heterosis in different crops or characteristics remains unclear.

Recently, with the development of modern biology and genomics, the study and description of the basis of crop heterosis at the molecular level have become a common topic for research and development. Heterosis genes related to rice tillering have been polymerized by Graded Pool-Seq and integrated genomic methods, and it has been reported that the dominance or incomplete dominance of heterozygous loci in rice plays an important role in the formation of heterosis^8–10^. Simultaneously, the technique of proteomics provides a novel research idea for analyzing the molecular mechanisms of heterosis. Leonardi et al.^11^ reported that protein quantitative polymorphism and non-additive genetic effects were related to heterosis. The production of heterosis in maize is affected by a variety of protein expression patterns, among which dominant expression pattern was the main^12–14^. However, none of the existing studies has used proteomics to explain the heterosis of tobacco.

Nicotine is a unique alkaloid in the tobacco plant, which offers great value in several fields, including the cigarette industry, medicine, and agriculture^15, 16^. Nicotine is a secondary metabolite transported over long distances, and the process of synthesis involves the formation of the pyridine ring and pyrrole ring, and the combination of the two rings^17^. For the pyrrole ring formation, putrescine is first directly synthesized from ornithine by ornithine decarboxylase (*ODC*)^18, 19^, or indirectly from arginine by arginine decarboxylase (*ADC*)^20^. Following, putrescine is catalyzed by putrescine-N-methyltransferase (*PMT*) to form N-methylputrescine, which was then catalyzed by N-methylputrescine catalytic enzyme (*MPO*) to form 4-methylaminobutyl ether^21, 22^. And wherein *PMT* is the rate-limiting enzyme for the synthesis and metabolism of nicotine^23^. For the formation of the pyridine ring, *QPT* is the rate-limiting enzyme for the synthesis and metabolism of nicotine^24^. Some studies have demonstrated that Isoflavone reductase-like protein (*A622*) and Berberine bridge enzyme-like protein (*BBL*) play a linking role in the combination of the pyridine and pyrrole ring to form nicotine^25, 26^.

Since the 1950s, tobacco heterosis has been studied and utilized in China^7, 27^. About 80% of the characteristics in the first generation of flue-cured tobacco hybrids showed different degrees of heterosis^28^, with the heterosis varying greatly in different characteristics and hybrid combinations, and most of the characteristics showed positive heterosis^29^. However, the molecular mechanism of nicotine heterosis in tobacco remains unclear, and there is no study utilizing proteomics to analyze the mechanism of formation of nicotine heterosis. Therefore, this study aimed to analyze the expression of different proteins in the parent lines and their hybrids to understand this mechanism.

## Results

### Heterosis performance of nicotine characters in tobacco hybrids

**Figure 1:**
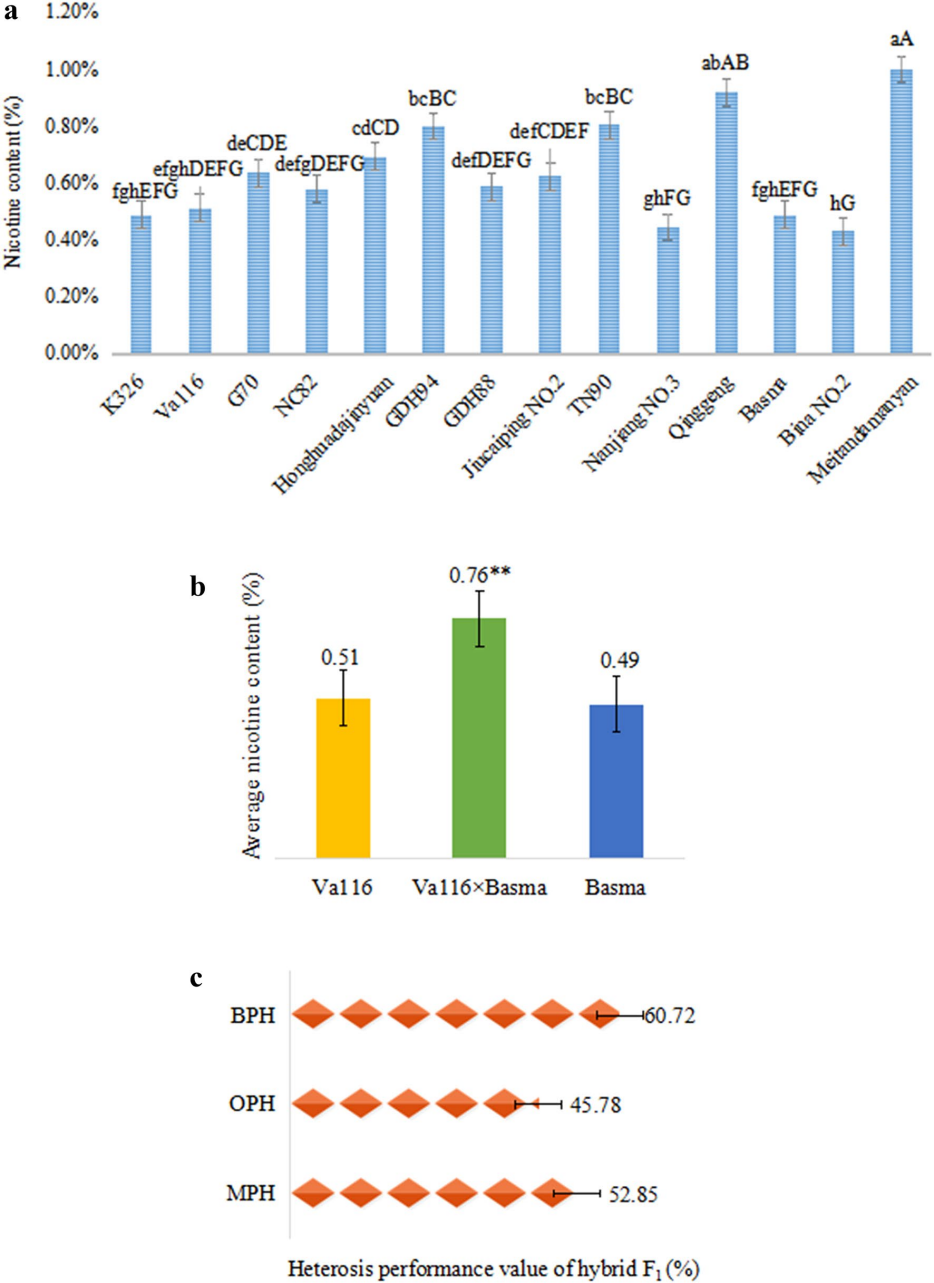
The characteristics of nicotinine and nicotinine heterosis in different materials. The letters and “*” represent significant differences. Significant differences among the nicotine content at *P* < 0.05 and *P* < 0.01 were determined by the Duncan's new multiple range test. (**a**) The difference of nicotine content in different parent materials. The lowercase letters represents significant difference (*P* < 0.05); and capital letters represents extremely significant difference (*P* < 0.01). (**b**) The difference of nicotine content in tobacco hybrids and their parents. “**” represents extremely significant difference (*P* < 0.01). (**c**) Heterosis performance value of nicotine content in hybrid F_1_. The OPH, MPH and BPH represents over high-parent heterosis, mid-parent heterosis, and below low-parent heterosis respectively. Figures generated using Microsoft Excel 2016.

In order to explore the heterosis of nicotine content in the leaves of tobacco hybrids, 11 materials with different nicotine contents were selected as parents and matched with hybrid combinations. There were significant differences in nicotine content between parents (Fig. 1a). The nicotine content in the different types and varieties of tobacco showed diversity, and the genetic resources for the improvement of nicotine content were abundant. The heterosis over mid-parent value reached a significant or extremely significant level among the different hybrid combinations (Supplementary Table 1), which informed us the existence of genetic differences in the nicotine heterosis among the selected parents. Combined with the previous research results of our laboratory^30, 31^, it was found that Va116 × Basma frequently showed a strong heterosis (Fig. 1b,c). The nicotine content of the hybrids was 0.76%, which was significantly higher than that of the two parent lines. The dominant value of mid-parent was 52.85%, the heterosis of super-parent and low-parent was both positive, and the latter was as high as 60.72%, indicating that nicotine content had a strong ability of transgression in hybrid offspring. Subsequently, we investigated the field agronomic traits of the hybrids and the parents (Table 1), and found that the hybrid Va116 × Basma performed significantly better over the two parents. We speculated about good plant growth potential provides an effective power for the synthesis, transport and accumulation of nicotine. Therefore, in this study, Va116 × Basma, Va116 and Basma were used as experimental materials for subsequent proteomic analyses to explore the mechanism of nicotine content heterosis in tobacco leaves.

**Table 1:** Field performance of agronomic traits of parents and hybrid (unit: cm).

Materials	Traits			
	Stem girth	Plant height	Leaf length	Leaf width
Va116	8.80 bB	79.03 cC	51.33 bB	25.42 bB
Va116 × Basma	11.11 aA	101.36 aA	70.36 aA	30.50 aA
Basma	8.74 bB	87.95 bB	55.94 bB	22.50 bB

The lowercase letters represents significant difference (*P* < 0.05); and capital letters represents extremely significant difference (*P* < 0.01).

### The difference in protein expression between hybrids and their parents

To determine the differences in expression characteristics of nicotine heterosis at the protein level, root tip tissues from three genotype materials were analyzed by DIA technology. Following quality evaluation and database comparison, 5346 proteins were identified from 28,042 peptide segments, and each protein contained at least one peptide segment. In the subsequent analysis, we quantified 4395 proteins in three genotypes simultaneously. The complete peptide fragments and protein matching information of these 4395 proteins are summarized in attachment 1.

**Figure 2:**
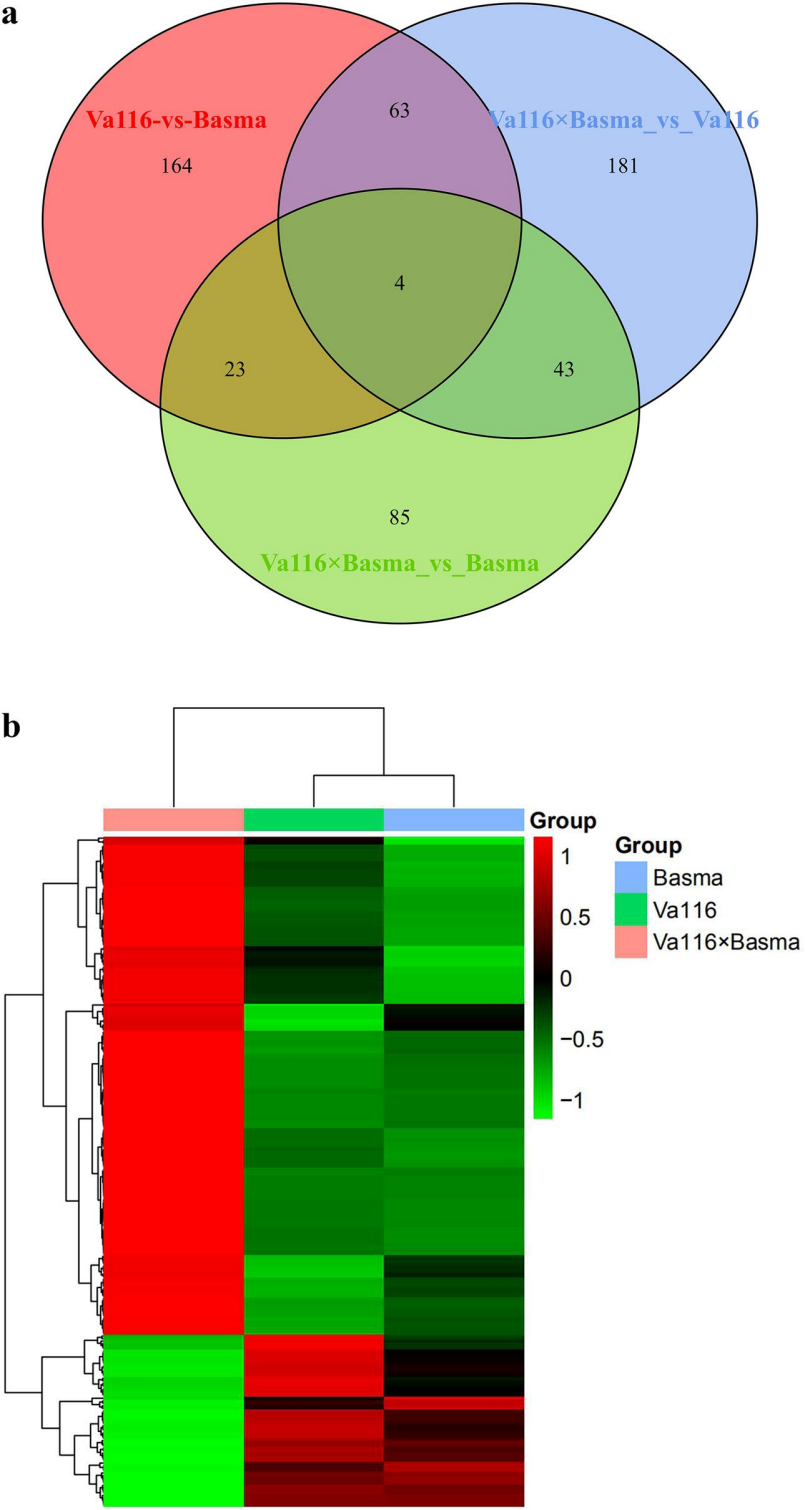
Venn diagram comparison and hierarchical cluster analysis of diferentially expressed proteins among genotypes. (**a**) Venn diagram comparison of diferential expressed proteins between the hybrid and its parents. (**b**) Hierarchical cluster analysis of diferentially expressed proteins among genotypes. Te color key represents protein expression quantity. Red indicates high relative expression and green indicates low relative expression. Figures generated using the web of http://www.bioinformatics.com.cn/.

To identify the differentially expressed proteins (DEPs) between hybrids and their parent inbred lines, a two-tailed T-test was conducted to test the significance of differences of the identified proteins between the genotypes. At *P* < 5%, 697 (15.86%, n = 4395) proteins showed significant differences among the three genotypes. Among these proteins, 254 proteins in Va116 and Basma showed significant differences in upregulation and downregulation, indicating a certain genetic distance between the two parental lines. Comparing the hybrids with their male and female parents, it was observed that 291 proteins (179 upregulated and 112 downregulated proteins) showed significant differences between Va116 × Basma and Va116, and 155 proteins (95 upregulated and 60 downregulated proteins) showed significant differences in Va116 × Basma and Basma (Table 2 and Supplementary Fig. 1). These DEPs might collectively account for heterosis in the nicotine content of tobacco hybrids. Analysis of the Venn diagrams indicated that the expression of 23.38% (63 and 4) of DEPs in Va116 and Basma was different to that in Va116 × Basma and Va116, whereas 10.63% (23 and 4) of DEPs were differentially expressed with Va116 × Basma and Basma, and only four DEPs co-existed between these three groups (Fig. 2a). The results indicated that the expression of proteins was generally differentially expressed, and there were significant differences between the different genotypes. We further explored the hierarchical clustering of differentially expressed proteins among the three genotypes and observed that the expression pattern of DEPs in Va116 × Basma showed a contrasting trend to that of the two parental lines (Fig. 2b).

**Table 2:** Statistical analysis of the quantitative results of differential proteins.

Comparison groups	Up		Down		Total	
	Number	Percentage	Number	Percentage	Number	Percentage
Va116-VS-Basma	141	3.21%	113	2.58%	254	5.78%
Va116 × Basma-VS-Va116	179	4.07%	112	2.54%	291	6.62%
Va116 × Basma-VS-Basma	95	2.16%	60	1.37%	155	3.53%

### Identification of DEPs in hybrids

**Figure 3:**
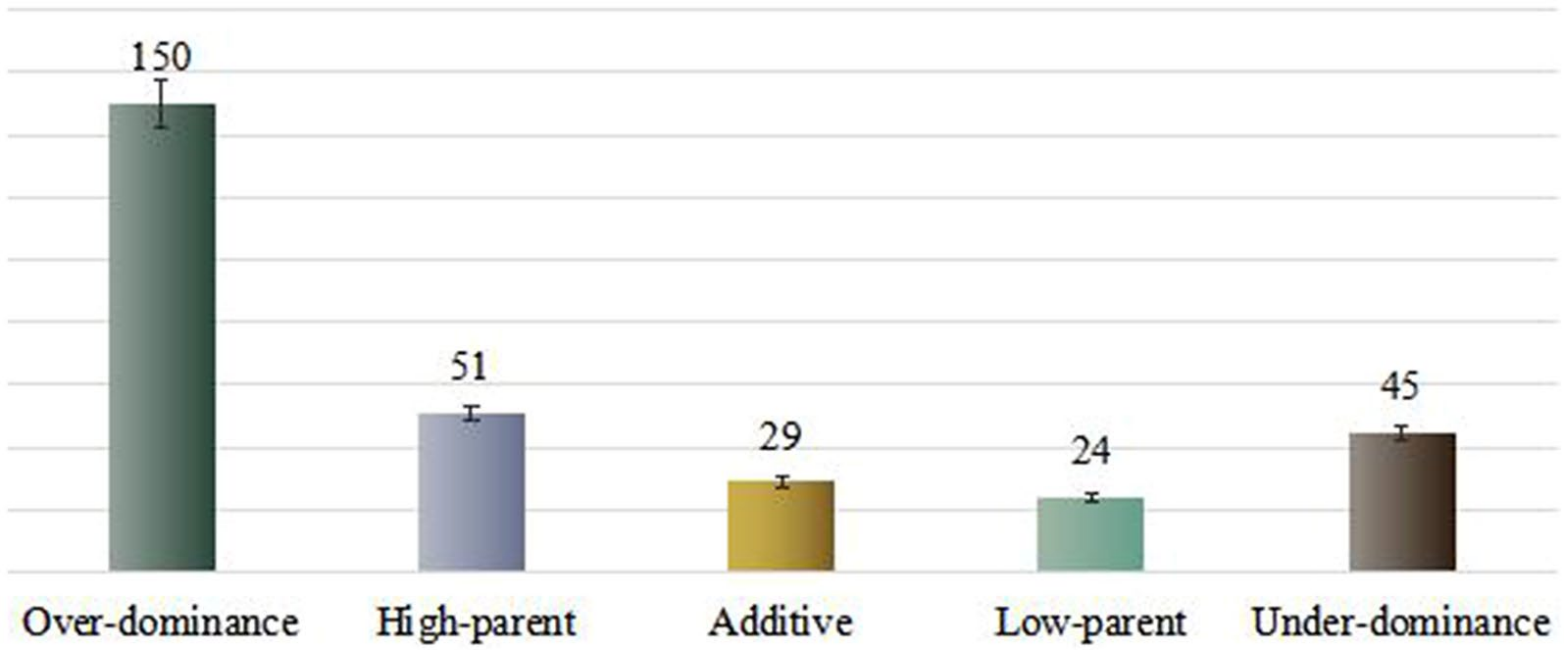
Classification of expression patterns of different proteins in hybrid. Figures generated using Microsoft Excel 2016.

To explore the mechanism of formation of nicotine heterosis in tobacco, the expression patterns of DEPs were classified to determine the effect of the expression patterns of additive and non-additive proteins on heterosis. In the hybrid Va116 × Basma, expression patterns of only 29 DEPs were close to the parental level, showing additive accumulation, whereas the remaining 270 (90.3%) DEPs showed non-additive accumulation (Supplementary Table 2), suggesting that the formation of nicotine heterosis was more influenced by the non-additive accumulation proteins. The over-dominant expression pattern accounted for 72.2% (150 and 45), while the dominant expression pattern accounted for only 27.8% (51 and 24) of the non-additive expressed proteins (Fig. 3). These results indicated that the over-dominant effect of the level of protein expression played a crucial role in the formation of nicotine heterosis. The upregulated DEPs accounted for a majority (76.9%, n = 195) of the over-dominant expressed proteins (Supplementary Table 2). It was further suggested that an increase in the expression of DEPs in hybrids could promote the heterosis of nicotine content traits.

### Functional enrichment analysis of non-additive expressed proteins

**Figure 4:**
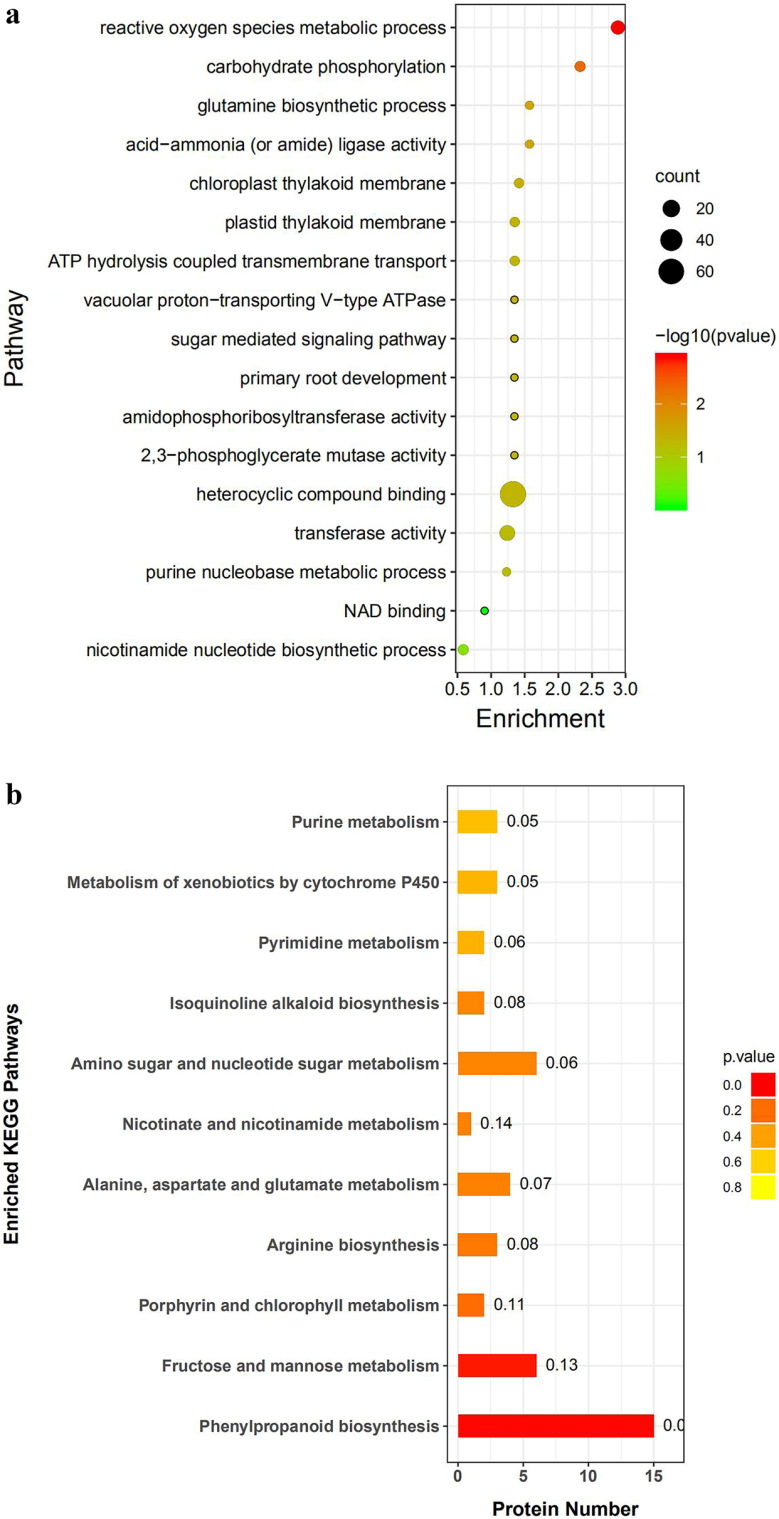
Enrichment analysis of non-additive expressed proteins. (**a**) GO Terms enrichment analysis of non-additive expressed proteins in hybrids and parents. (**b**) KEGG annotation pathway analysis of non-additive expressed proteins in hybrids and parents. Figure generated using RStudio software loaded with the ggplot package.

Some studies have demonstrated that non-additive expressed proteins play a vital role in the performance of offspring hybrids. To understand the biological effects of such proteins, GO and KEGG functional enrichment analysis was performed on the DEPs (Fig. 4a,b, attachment 2 and 3). A majority of the DEPs were enriched in terms of binding to heterocyclic compounds, transferase activity, and active oxygen metabolism. The upregulated over-dominant expressed proteins were significantly enriched in heterocyclic compound binding, primary root development, photosynthesis, amide phosphoribosyltransferase activity, and amide ligase activity. The upregulated dominant expressed proteins were enriched in the biosynthesis of nicotinamide. KEGG analyses of F_1_ hybrids also indicated that three DEPs were involved in arginine metabolism, four were associated with aspartic acid and glutamate metabolism, 15 were related to phenylpropane metabolism, six were involved in amino sugar and nucleotide metabolism, and one DEP was involved in the nicotinic acid and nicotinamide metabolism pathway. To summarize, these DEPs played an important role in the synthesis and metabolism of tobacco alkaloids, and their super-parental expression in hybrid offspring was beneficial to the performance of nicotine traits, thus showing strong heterosis.

### qRT-PCR of genes related to nicotine synthesis and transport in hybrids

**Figure 5:**
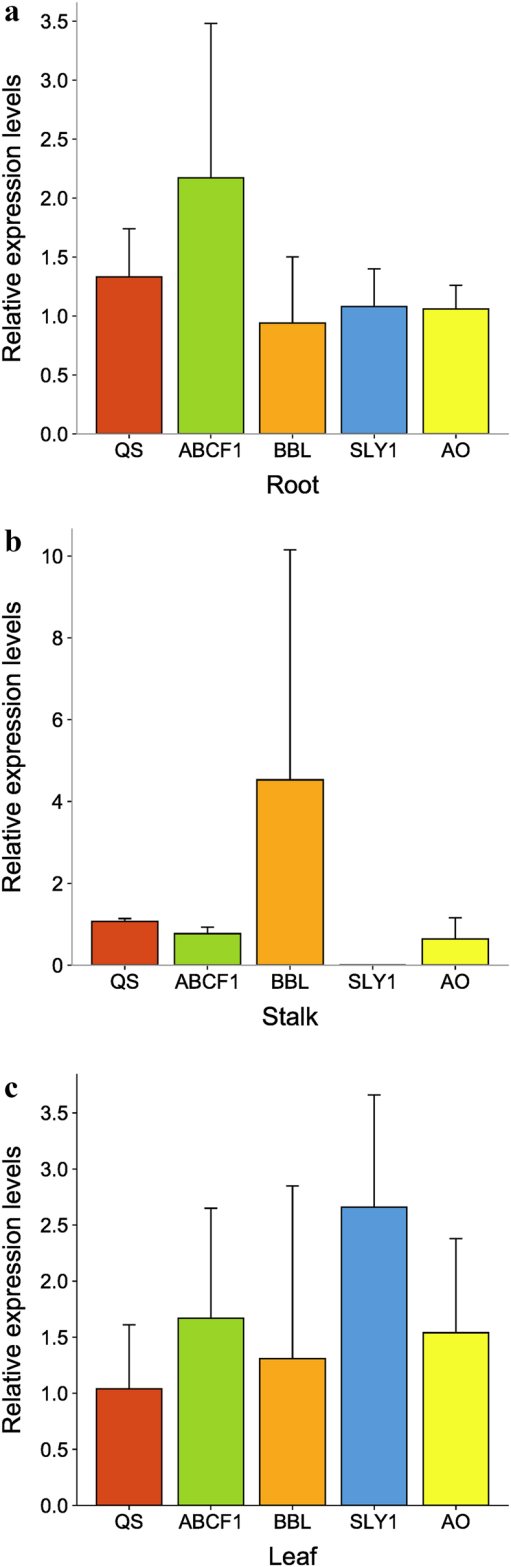
Relative expression Levels of genes related to nicotine synthesis and transport in hybrids. The (**a**–**c**) represents the relative expression of the gene in the three organs, root, leaf and stalk, respectively. The relative expression levels of hybrids was relative to the mid-parent value. Figures generated using the web of http://www.bioinformatics.com.cn/.

To validate the expression quantity of vital DEPs obtained by DIA proteomics, quantitative real-time PCR (qRT-PCR) was used to investigate their expression profiles at mRNA level in the roots, stalk and leaf, respectively. We confirmed that both the *QS*, *ABCF1*, *BBL*, *SLY1* and *AO* genes were upregulated expression in hybrids (Fig. 5a–c), and the selected five genes had expression specificity in the different organs. *BBLs*, a key factor linking pyridine and pyrrorings in nicotinic synthesis, showed the higher-parent expression pattern in Va116 × Basma both in root, stalk and leaf. The higher-parent expression of *ABCF1* and *SLY1* in the root and leaf further illustrateed that their possible function as nicotinic transport carriers. The expression patterns of genes related to nicotine synthesis and transport at mRNA level even more verified the results of proteomics experiments that the synthesis and transport capacity of nicotinoids in hybrids is enhanced.

## Discussion

With the development of quantitative genetics^32^, molecular genetics (Liu et al., 2011) and genomics^33^, experimental methods such as DNA labeling technology^34^, RNA sequencing^35^, metabolomics^36^, and proteomics^37^ have been improved. It has become a common trend to study and describe the basis of formation of crop heterosis from the perspective of molecular regulation^38, 39^. Proteomics has been regarded as the main part of functional genomics, with significant application in the analyses of the mechanism of heterosis. A comprehensive differential proteomic analysis between the hybrid Va116 × Basma with strong heterosis in nicotine content and its parent lines Va116 and Basma was conducted. A total of 4395 differentially expressed proteins were detected in all the three genotypic materials, of which 697 showed significant differences. Compared with previous studies^40, 41^, the detection abundance and efficiency were significantly improved.

Comparing Va116 × Basma with its parental lines using DIA holographic scanning, 291 and 155 differential proteins were obtained, and most of them were upregulated. Based on the hierarchical clustering, the protein expression profile of hybrids showed a contrasting trend to that of the two parental lines. As for the number of differential proteins, the protein expression profile of hybrid Va116 × Basma was more like that of the male parent Basma, indicating that the male parent contributed more to the formation of traits of the hybrid offspring. An increasing number of studies have reported that the proteins of the hybrid offspring showed an additive expression pattern^42^. The over-dominant effect observed in this study was consistent with the conclusions of^43^ and Ellen Moura et al*.*^44^. The findings were also in line with our previous research at the transcription level^45^. Collectively, the non-additive expressed proteins synergistically produced heterosis of tobacco phenotypes.

During the formation of the pyrrole ring, arginine is the most direct and important donor of putrescine^46^. Aspartic acid is an effective precursor of nicotinic acid formation in the pyridine ring^47^. The KEGG analysis in this study revealed that the synthetic metabolic pathways of arginine and aspartic acid were significantly enriched in the hybrids, and several over-dominant proteins were involved. Previous studies have reported that glutamine synthetase facilitated nicotine synthesis by promoting nitrogen assimilation^48^. The key to nicotine synthesis lies in the combination of the pyridine ring and pyrrole ring. Several proteins involved in the binding of heterocyclic compounds were identified via GO analysis. Among them, the BBL protein had been previously demonstrated to play an important role in the final stages of nicotine synthesis, when the pyridine ring and pyrrole ring are linked together^49^. Furthermore, the developmental pathway of primary roots was significantly enriched, in which non-specific serine/threonine protein kinase and replication factor C subunit 1 were significantly up-regulated in hybrids, indicating that the roots of hybrids had stronger resistance to cell senescence and apoptosis, as well as improved ability to repair the damage. All these indicate that hybrid progenies of tobacco had stronger nicotine synthesis ability.

We also identified several differential proteins that could be related to nicotine transport. Among these proteins, glutathione S-transferase exists in vacuoles and participates in transmembrane and intracellular transport, which has been demonstrated in previous studies to be related to anthocyanin transport and some natural hormone IAA transport carriers^50^. The enzyme-like family members of ABC transporter C and F, and *SLY1*-like *SEC1* family transporter, had been identified as transmembrane transporters in previous studies^51, 52^. Xie^53^ reported that the *ABCG* subfamily gene *Nt WBC13* had a strong nicotine transport capacity, but for the C and F family were not reported. The expressions of these three proteins were observed to be significantly upregulated in hybrids, leading to the hypothesis that the increase of nicotine content in hybrids may be induced and promoted by these transporters, due to which the efficiency in nicotine transport and accumulation in hybrids is also improved.

We used tobacco roots for transcriptome analysis and observed that some genes involved in nicotine synthesis and transport, such as *NUP1, 2*, *JAT2*, and *Mate1,2*, were significantly upregulated in the roots of hybrids^45^. However, no significant upregulation or downregulation of the corresponding proteins of these genes was observed at the protein level, which agrees with the conclusion of Steinmetz et al.^54^ that there was a certain difference between the changes at the intracellular mRNA and protein level. The protein is the ultimate embodiment of life activities, which is closely related to the characteristics of organisms. Therefore, it is proposed that several proteins with transport functions identified in this study are directly involved in the transport and accumulation of nicotine. However, further experiments are required to determine whether glutathione S-transferase, *ABC* transporter family C and F family members, and *SEC1* family transporter *SLY1* can participate in nicotine transport.

In conclusion, our research has provided a novel perspective for the analysis of the mechanism of nicotine heterosis at the protein level. Moreover, nicotine is an important secondary metabolite in tobacco, and its synthesis and transport are influenced by multiple biochemical reactions and metabolic pathways, including photosynthesis, glycometabolism, nitrogen metabolism, and TCA cycle. Any factors related to the growth or development of source-sink-translocation will affect the synthesis and accumulation of nicotine. The over-dominant accumulation effect of these differentially expressed proteins may be a critical reason for the strong dominance of nicotine content in hybrids.

## Methods

### Plant material and analysis of nicotine traits

The F_1_ tobacco hybrid of Va116 × Basma and both the parent seeds (Va116: flue-cured tobacco; Basma: oriental tobacco) selected in this study were provided by the Institute of Tobacco, Chinese Academy of Agricultural Sciences. We guarantee that the collection of plant material and experimental research and field studies on plants comply with relevant institutional, national, and international guidelines and legislation. The field experiment was adopted randomized block design, with three biological replicates, and conducted at the tobacco research experimental base of Guizhou University during 2018 and 2019. Setting three repetitions and experimental units were sixty plant rows with intra-row plant spacing and row spacing of 55 and 110 cm, respectively. All plants were topped on the same day after greater than 50% of the plants flowered (68th days after transplanting). Samples were collected on the 75th day after transplantation. The middle leaves and stalk samples at the 9–11 leaf position and young roots were selected, and three normal-growing plants were randomly selected for mixed sampling in each plot. Fresh samples of roots, stalks and leaves were used for RT-qPCR experiments, in addition, the fresh samples of root tips were also used for proteomics analysis. The leaf samples were used for chemical analyses. The root tip samples were evenly mixed in equal quantities, washed with clear water, and rinsed with PBS. Later, the samples were placed in different sterilized centrifuge tubes frozen with liquid nitrogen and immediately stored in a refrigerator at –80 °C. The frozen preservation method of leaf and stalk samples was the same as that of roots. The enzymes in the leaf samples were denatured at 105 °C for 30 min, after which the samples were dried at 75 °C, ground into powder, bagged, and sealed for storage. According to the method of Shoji et al.^55^, nicotine was separated from the extract of dry leaf samples and analyzed using gas chromatography.

### Preparation of protein samples and determination of protein concentration

The protein samples were prepared by the TCA-acetone precipitation method. A 100% (w/w) trichloroacetic acid (TCA) solution was prepared with 500 g TCA (ex-factory), dissolved in 350 ml dH_2_O, and stored at room temperature. One part of the TCA reserve solution was added to four parts of protein samples and incubated at 4 °C for 10 min. The protein samples were centrifuged at 14,000 rpm for 5 min in a microcentrifuge. Then, the supernatant was removed to aggregate the protein precipitate. The precipitate was washed with 200 µL of cold acetone and then centrifuged at 14,000 rpm for 5 min in a tuner. Afterwards, the precipitate was washed twice with acetone. The test tube was dried in a heating block at 95 °C for 5–10 min to remove acetone, thereby drying the precipitate. For SDS-PAGE, 2X or 4X sample buffer (with or without bME) was added and the samples were boiled in a 95 °C heating block for 10 min before loading the sample onto the polyacrylamide gel.

After the quantification of the protein, an appropriate amount of protein was taken and mixed into a pooled sample (about 200 µg-400 µg), which was used to construct the Spectral Library. About 20 µg protein from each original sample was tested by SDS-PAGE to evaluate the consistency between samples. The protein concentration was determined using a BCA protein concentration determination kit, according to the manufacturer’s instructions.

### Protein enzymolysis and mass spectrometry

Protein enzymolysis was carried out according to the methods of Li^56^ and Xu^57^. The concentration of peptide fragments was determined at OD_280_. About 100 µg of the peptide fragments with low abundance protein after high abundance separation were classified with HPRP, and all the components were collected. The freeze-dried peptides of each component were reconstituted with 10 µL of 0.1% FA, and the peptide concentration was determined at OD_280_. Then, 2 µg of peptide fragments were taken out sequentially, and an appropriate amount of the iRT standard peptide fragments were added for DDA mass spectrometry, and each component was analyzed using mass spectrometry for 90 min. The chromatographic separation was conducted using an HPLC system (Easy nLC-1200). Mass spectrometry was performed according to the methods described by Zhou^58^, Li^59^, and Cui^60^.

### Processing and analysis of proteomics data

The DDA data were searched using the program Maxquant (Maxquant_1.5.3.17). The database was downloaded from tobacco_uniprot, and the sequence of the iRT peptide fragment was added to the database. The parameters were set according to the protocols described Fu^61^ and Shao et al.^62^. The original raw files and the search results were exported to the software Spectronaut (Spectronaut Pulsar Xerox 12.0.20491.4) to build the Spectral Library. The software parameters were set as follows: retention time prediction type was set to dynamic iRT, interference on MS2 level correction was enabled, and cross run normalization was enabled. All the results were filtered by setting a parameter Q value cutoff of 0.01 (equivalent to FDR < 1%).

### Bioinformatics analyses

In this study, a hierarchical clustering algorithm was used to analyze the differentially expressed proteins in the compared groups. The package ComplexHeatmap R (R Version 3.4) was used to classify the two dimensions of sample and protein expression simultaneously (distance algorithm: Euclid, connection mode: Average linkage) and generate a hierarchical clustering heat-map. The GO function of the identified protein was annotated using the software Blast2Go (https://www.blast2go.com/). Then, Fisher's exact test was carried out for the GO functional enrichment analysis of differentially expressed proteins. The KEGG pathway of the target protein set was annotated by the software KAAS (KEGG Automatic Annotation Server). The KEGG Mapper tool (http://www.genome.jp/kegg/mapper.html) was used to create KEGG pathway maps.

### Identification and classification of differentially expressed proteins

A one-way ANOVA (FDR-adjusted p-value of 0.05) with a two-tailed test was conducted to detect the differentially expressed proteins between hybrids and parental lines. A p-value ≤ 0.05 indicated significant differences in the median values between hybrids and their parents. A Fold change value < 1.5 or > 0.67 indicated an additive expression protein, whereas a Fold change value ≥ 1.5 or ≤ 0.67 indicated a non-additive expression protein. Non-additive expressed proteins could be divided into more specific categories. The ratio of the expression levels of hybrid to the high expressing parent lines was 10% higher than the threshold value of 1.5 (Over-dominant expression). The ratio of the expression level of hybrids to the high expressing parental lines was lower than 10% of the threshold and greater than 1.5. The ratio of the expression level of the hybrid to the high expressing parent line was 10% lower than the threshold (0.67) (Under-dominant expression). The ratio of the expression level of hybrid to the high expression parent lines was 10% higher than the threshold and less than 0.67 (Low-parent expression)^63^.

### Real-time fluorescence quantitative PCR

To validate the amount of protein expression obtained by DIA proteomics, five proteins(*QS, ABCF1, BBL, SLY1 and AO*) associated with nicotinic synthesis and transport were randomly selected for real-time fluorescence quantitative PCR (RT-qPCR) experiments. Experiments performed with the SYBR Premix Ex Taq kit (Takara). Applied Biosystems 7500 Real-Time PCR system (Life Technologies Corporation, Beverly, MA, USA). The protein and corresponding primers for the qPCR test were listed in Supplementary Table 3. To calculate the relative expression level of individual gene, 2^−ΔΔCt^ method^64^ was adopted.

### Statistical analyses

Duncan's new multiple range test was used to analyze the variation in nicotine content (*P* < 0.05) using the software SPSS (version 16.0). The values of over high-parent heterosis (OPH), mid-parent heterosis (MPH), and below low-parent heterosis (BPH) were calculated according to the following formula: ${\text{OPH}}\,{\text{(\% )}} = \left( {\frac{{{\text{F}}1 - {\text{HP}}}}{{{\text{HP}}}}} \right) \times 100$, ${\text{MPH}}\,{\text{(\% )}} = \left( {\frac{{{\text{F}}1 - {\text{MP}}}}{{{\text{MP}}}}} \right) \times 100$, ${\text{BPH}}\,{\text{(\% )}} = \left( {\frac{{{\text{F}}1 - {\text{LP}}}}{{{\text{LP}}}}} \right) \times 100$, where F_1_ represents the first hybrid generation, HP represents the high-value parent, MP represents the average parent value $\left( {\frac{{{\text{parent}}1 + {\text{parent}}2}}{2}} \right)$, and LP represents the low-value parent.

## Supplementary Information

Supplementary Information 1.

Supplementary Information 2.

Supplementary Information 3.

Supplementary Information 4.

## Data Availability

All data generated or analyzed during this study are included in this published article and its supplementary information files.

## References

[1] Tester M, Langridge P (2010). Breeding technologies to increase crop production in a changing world. Science.

[2] Birchler J, Auger L, Riddle N (2003). In search of the molecular basis of heterosis. Plant Cell.

[3] Schnable P, Springer N (2013). Progress toward understanding heterosis in crop plants. Annu. Rev. Plant Biol.

[4] Cheverud JM, Routman EJ (1995). Epistasis and its contribution to genetic variance components. Genetics.

[5] Minvielle F (1987). Dominance is not necessary for heterosis: a two-locus model. Genet. Res..

[6] Wan J (2018). Genetic crop improvement: a guarantee for sustainable agricultural production. Engineering.

[7] Wang S, Xu L, Fu XK, Jian X, Wu B (2005). Present situation and prospect of heterosis utilization in Flue-cured tobacco in China. Chin. Tobacco Sci..

[8] Wang C (2019). Dissecting a heterotic gene through GradedPool-Seq mapping informs a rice-improvement strategy. Nat. Commun..

[9] Huang X (2016). Genomic architecture of heterosis for yield traits in rice. Nature.

[10] Huang X (2015). Genomic analysis of hybrid rice varieties reveals numerous superior alleles that contribute to heterosis. Nat. Commun..

[11] Leonardi A, Damerval C, Hebert Y, Gallais A, De Vienne D (1991). Association of protein amount polymorphism (PAP) among maize lines with performances of their hybrids. Theor. Appl. Genet..

[12] Dahal D, Newton KJ, Mooney BP (2016). Quantitative proteomics of Zea mays hybrids exhibiting different levels of heterosis. J. Proteome Res..

[13] Song X (2007). Wheat (*Triticum aestivum* L.) root proteome and differentially expressed root proteins between hybrid and parents. Proteomics.

[14] Song X, Ni Z, Yao Y, Zhang Y, Sun Q (2009). Identification of differentially expressed proteins between hybrid and parents in wheat (*Triticum aestivum* L.) seedling leaves. Theor. Appl. Genet..

[15] Chen J, Liu J, Long H (2004). Study on mineral nutrition and Main chemical Composition content characteristics of Chinese tobacco leaf. Chin. Tobacco.

[16] Xue X, Chen Y, Wang M, Pan W, Li J (2008). Research status of nicotine. Anhui Agric. Sci..

[17] Baldwin IT (1999). Inducible nicotine production in native Nicotiana as an example of adaptive phenotypic plasticity. J. Chem. Ecol..

[18] Imanishi S (1998). Differential induction by methyl jasmonate of genes encoding ornithine decarboxylase and other enzymes involved in nicotine biosynthesis in tobacco cell cultures. Plant Mol. Biol..

[19] Marton LJ, Pegg AE (1995). Polyamines as targets for therapeutic intervention. Annu. Rev. Pharmacol. Toxicol..

[20] Tiburcio AF, Galston A (1985). Arginine decarboxylase as the source of putrescine for tobacco alkaloids. Phytochemistry.

[21] Kutchan TM (1995). Alkaloid biosynthesis [mdash] the basis for metabolic engineering of medicinal plants. Plant Cell.

[22] Chou WM, Kutchan TM (1998). Enzymatic oxidations in the biosynthesis of complex alkaloids. Plant J..

[23] Saunders JW, Bush LP (1979). Nicotine biosynthetic enzyme activities in Nicotiana tabacum L. genotypes with different alkaloid levels. Plant Physiol..

[24] Wagner R, Wagner K (1985). The pyridine-nucleotide cycle in tobacco Enzyme activities for the de-novo synthesis of NAD. Planta.

[25] Deboer KD, Lye JC, Aitken CD, Su AK-K, Hamill JD (2009). The A622 gene in Nicotiana glauca (tree tobacco): evidence for a functional role in pyridine alkaloid synthesis. Plant Mol. Biol..

[26] Kajikawa M, Hirai N, Hashimoto T (2009). A PIP-family protein is required for biosynthesis of tobacco alkaloids. Plant Mol. Biol..

[27] Ai S (1987). Research and utilization status of tobacco hybrid generation. Chinese tobacco.

[28] Tong D (1997). Tobacco Breeding.

[29] Wu S (2001). Heterosis and genetic analysis of several agronomic characters of flue-cured tobacco. Chin. J. Tobacco.

[30] Wang GQ (2015). Analysis of Tobacco Nicotine Heterosis and Its Gene Differential Expression.

[31] Long Y (2016). Construction of Tobacco Subtractive cDNA Library and Sequence Analysis of Nicotine-related EST.

[32] Chen Z (2020). Quantitative genetics theory of heterosis and related application in maize breeding. Maize Science.

[33] Jia, J. & Li, Y. Plant Genomics and Gene Discovery in Germplasm Resources. *Chinese Agricultural Science* **37**, Chinese Agricultural Science (2004).

[34] Chen X, Gao Z (2019). The study and application of DNA molecular marker technique. Mol. Plant Breed..

[35] Wang Z, Gerstein M, Snyder M (2009). RNA-Seq: a revolutionary tool for transcriptomics. Nat. Rev. Genet..

[36] Hozzein WN, Blumenberg M (2020). Metabolomics: New Insights into Biology and Medicine.

[37] Jesus VJ-N, Luis V, Mari AC, Maria DR (2020). Plant Proteomics.

[38] Liu Z, Jiang J, Yang H, Jiang X, Li J (2019). Research advance of plant heterosis. Mol. Plant Breed..

[39] Zhao Y, Qin Z, Zhou X, Xin M (2018). Research advance of plant heterosis. Northern Horticult..

[40] Li H (2007). Proteome Analysis of Root Tip Tissue Difference Before and After Curing Flue-Cured Tobacco.

[41] Lin S (2012). Comparison of preparation methods of tobacco root protein by two - dimensional electrophoresis. Guizhou Agricult. Sci..

[42] Xing J, Sun Q, Ni Z (2016). Proteomic patterns associated with heterosis. Biochimica et Biophysica Acta (BBA)-Proteins Proteom..

[43] Guo B (2013). Comparative proteomic analysis of embryos between a maize hybrid and its parental lines during early stages of seed germination. PLoS ONE.

[44] Vale EM (2016). Comparative proteomic analysis of the heterosis phenomenon in papaya roots. Sci. Hortic..

[45] Tian M (2018). Transcriptomic analysis reveals overdominance playing a critical role in nicotine heterosis in *Nicotiana tabacum* L. BMC Plant Biol..

[46] Satriano J (2004). Arginine pathways and the inflammatory response: interregulation of nitric oxide and polyamines. Amino Acids.

[47] Jackanicz TM, Byerrum RU (1966). Incorporation of aspartate and malate into the pyridine ring of nicotine. J. Biol. Chem..

[48] Chen D (2009). The Effect of Tobacco Root Tip Tissue Specific Proteins on Nicotine Biosynthesis was Investigated.

[49] Kajikawa M, Shoji T, Kato A, Hashimoto T (2011). Vacuole-localized berberine bridge enzyme-like proteins are required for a late step of nicotine biosynthesis in tobacco. Plant Physiol..

[50] Sandermann H (1994). Higher plant metabolism of xenobiotics: the'green liver'concept. Pharmacogenetics.

[51] Zhao X (2012). Function Analysis of SM Protein AtSLY1 in Arabidopsis Protein Transport.

[52] Wang X (2017). Research progress of ABC transporters in Arabidopsis thaliana. J. Plant Physiol..

[53] Xie X (2014). Identification of ABC Transporter Family in Tobacco and Study on the Function of Secondary Metabolite Transport.

[54] Steinmetz LM, Davis RW (2004). Maximizing the potential of functional genomics. Nat. Rev. Genet..

[55] Shoji T (2009). Multidrug and toxic compound extrusion-type transporters implicated in vacuolar sequestration of nicotine in tobacco roots. Plant Physiol..

[56] Li S (2019). Study on the Formation Mechanism of Yak Smooth Muscle Tenderness Based on Proteomics.

[57] Xu H (2019). Study on the Mechanism of Protein-Related Changes of Wall Polysaccharides in Different Growth Stages of Lycium barbarum l.

[58] Zhou, G. Analysis of differential expression of protein secreted by Banana Fusarium wilt. *South China Agricultural University*, 1-z (2016).

[59] Li C (2017). Study on Human Exposure and Related Markers of PCDD/Fs in Chlorination Industry Environment in Tianjin.

[60] Cui H (2018). Studies on the Function of the PiGK5 Gene and the Phosphorylated Proteomics Analysis of Sexual Reproduction Induced by 1 Sex Hormone in Phytophthora Species.

[61] Fu L (2019). Study on the Mechanism of Energy Metabolism Disorder During Cryopreservation of Human Sperm.

[62] Shao R, Xu H, Liu S, Wang X (2018). The salivary protein of bemisia tabaci was identified by lC-MS/MS. J. Environ. Entomol..

[63] Zhao P (2015). Phylogenetic Analysis of Long Internode Heterosis Under Ear of Maize.

[64] Livak K, Schmittgen T (2001). Analysis of relative gene expression data using real-time quantitative PCR and the 2(-Delta Delta C(T)) Method. Methods.

