# Supplementary material for: Effect of the over-dominant expression of proteins on nicotine heterosis via proteomic analysis
Source: Sci Rep. 2021 Oct 26;11:21063. doi: 10.1038/s41598-021-00614-x (PMC8548390; doi:10.1038/s41598-021-00614-x)
Supplement: Supplementary file 1 — Supplementary Information 1. [file 41598_2021_614_MOESM1_ESM.docx]

| Hybrid combinations names | Mid-parent heterosis(%) | Hybrid combinations names | Mid-parent heterosis(%) |
| --- | --- | --- | --- |
| Va116×Basma | 52.85aA | K326×TN90 | -1.11bcdefghABCD |
| K326×Nangjiang No.3 | 30.27abAB | Va116×GDH88 | -3.32bcdefghABCD |
| Va116×Bina No.1 | 25.01abcABC | Va116×Nangjiang No.3 | -3.36bcdefghABCD |
| K326×Bina No.1 | 21.76abcdABC | G70×K326 | -4.30bcdefghABCD |
| K326×Basma | 21.45abcdABC | K326×Qinggeng | -5.62bcdefghABCD |
| K326×Jiucaiping No.2 | 19.13abcdABCD | Honghuadajinyuan×TN90 | -10.33bcdefghBCD |
| Honghuadajinyuan×Basma | 17.72abcdeABCD | GDH94×TN90 | -10.67bcdefghBCD |
| K326×GDH88 | 16.63abcdeABCD | Honghuadajinyuan×Qinggeng | -10.71bcdefghBCD |
| NC82×K326 | 16.32abcdefABCD | GDH94×Basma | -10.93bcdefghBCD |
| Va116×Qinggeng | 15.10abcdefgABCD | G70×Meitandamanyan | -11.63bcdefghBCD |
| NC82×GDH88 | 14.52abcdefgABCD | G70×Nangjiang No.3 | -12.91bcdefghBCD |
| Honghuadajinyuan×GDH88 | 12.09abcdefgABCD | G70×TN90 | -13.41bcdefghBCD |
| Honghuadajinyuan×Jiucaiping No.2 | 10.13abcdefgABCD | Va116×Meitandamanyan | -13.76bcdefghBCD |
| GDH94×K326 | 10.05abcdefgABCD | GDH94×Nangjiang No.3 | -13.79bcdefghBCD |
| Honghuadajinyuan×Nangjiang No.3 | 9.23abcdefgABCD | K326×Meitandamanyan | -14.15bcdefghBCD |
| Va116×Jiucaiping No.2 | 7.50bcdefgABCD | NC82×TN90 | -16.82cdefghBCD |
| NC82×Basma | 5.49bcdefghABCD | GDH94×Meitandamanyan | -21.65defghBCD |
| Va116×TN90 | 4.14bcdefghABCD | GDH94×Jiucaiping No.2 | -23.41defghBCD |
| Honghuadajinyuan×Meitandamanyan | 2.99bcdefghABCD | NC82×Qinggeng | -27.91efghBCD |
| NC82×Jiucaiping No.2 | 2.74bcdefghABCD | G70×Qinggeng | -29.50fghBCD |
| G70×Basma | 2.39bcdefghABCD | G70×Jiucaiping No.2 | -30.04ghBCD |
| G70×GDH88 | 1.34bcdefghABCD | NC82×Meitandamanyan | -30.73ghCD |
| NC82×Nangjiang No.3 | -0.12bcdefghABCD | GDH94×Qinggeng | -39.70hD |
| GDH94×GDH88 | -0.4bcdefghABCD |  |  |

Supplementary table 1. The nicotinic content heterosis value of different hybrid combinations (%). The significant differences were determined by the Duncan's new multiple range test. The lowercase letters represents significant difference (P＜0.05); and capital letters represents extremely significant difference (P＜0.01).

| Proteins | Descriptions | Foldchange | P Value | Up or  Down |
| --- | --- | --- | --- | --- |
| Additive Expression | | | | |
| A0A0A8J4Z6 | Putative aminoacylase | 0.72053 | 0.00306 |  |
| A0A1S3WZZ3 | alpha-glucosidase-like | 0.76455 | 0.02829 |  |
| A0A1S3XCL4 | aspartate aminotransferase-like isoform X1 | 1.39468 | 0.04643 |  |
| A0A1S3XCY5 | 3-oxoacyl-[acyl-carrier-protein] synthase I, chloroplastic | 1.38320 | 0.00788 |  |
| A0A1S3XEH9 | 40S ribosomal protein SA | 0.74408 | 0.02921 |  |
| A0A1S3XFX9 | plasma membrane-associated cation-binding protein 1-like | 0.77142 | 0.03130 |  |
| A0A1S3XSB2 | GDSL esterase/lipase At5g37690-like | 1.41299 | 0.03000 |  |
| A0A1S3XSQ1 | 60S ribosomal protein L23 | 0.69872 | 0.01750 |  |
| A0A1S3XXP1 | NAD(P)H:quinone oxidoreductase-like isoform X1 | 0.75696 | 0.03973 |  |
| A0A1S3XYU0 | eukaryotic initiation factor 4A-3 | 1.49469 | 0.02152 |  |
| A0A1S3YKN0 | cell division protein FtsZ homolog 2-2, chloroplastic-like isoform X2 | 1.35870 | 0.04111 |  |
| A0A1S3YQ39 | ATP-citrate synthase alpha chain protein 2 | 1.38465 | 0.02959 |  |
| A0A1S3Z1M3 | heme-binding protein 2-like | 0.68494 | 0.02960 |  |
| A0A1S3ZLX6 | UDP-N-acetylglucosamine diphosphorylase 1-like | 1.31199 | 0.03939 |  |
| A0A1S3ZNG5 | trifunctional UDP-glucose 4,6-dehydratase | 1.46251 | 0.00668 |  |
| A0A1S3ZPV7 | Eukaryotic translation initiation factor 3 subunit K | 1.21459 | 0.03162 |  |
| A0A1S3ZUL4 | 26S proteasome non-ATPase regulatory subunit 14 homolog | 1.40045 | 0.00169 |  |
| A0A1S3ZWX9 | zinc finger CCCH domain-containing protein 67-like isoform X2 | 1.44850 | 0.02552 |  |
| A0A1S4A241 | Importin subunit alpha | 1.43299 | 0.02613 |  |
| A0A1S4ARU0 | Ubiquitin thioesterase | 0.72494 | 0.03773 |  |
| A0A1S4BCE3 | villin-4-like | 0.74936 | 0.03011 |  |
| A0A1S4BI71 | D-3-phosphoglycerate dehydrogenase | 1.41573 | 0.04065 |  |
| A0A1S4CTN8 | SUMO-activating enzyme subunit | 1.34292 | 0.03880 |  |
| A0A1S4CWR4 | probable prefoldin subunit 2 | 0.71078 | 0.02392 |  |
| A0A1S4CZ29 | putative amidase C869.01 | 0.70945 | 0.01201 |  |
| A0A1S4D3Y3 | Carboxypeptidase | 1.25736 | 0.02400 |  |
| A0A1S4DJI8 | Annexin | 0.69047 | 0.01426 |  |
| A0A1S4DNN5 | probable aspartyl aminopeptidase | 1.30303 | 0.02960 |  |
| Q05214 | Actin | 1.46870 | 0.02916 |  |
| Over-dominance Expression | | | |  |
| A0A1S4DKJ7 | calcium-binding protein 39-like | 38.57239 | 0.03516 | ↑ |
| A0A1S4B9L8 | protein ABHD11-like | 19.84328 | 0.03910 | ↑ |
| A0A1S3Y3U0 | Histone H2A | 9.50873 | 0.03308 | ↑ |
| Q9SBZ6 | Uncharacterized protein Sar8.2j | 7.41555 | 0.00099 | ↑ |
| A0A1S4BCX2 | glutathione S-transferase U8-like | 7.31033 | 0.00170 | ↑ |
| A0A1S3YT76 | heat stress transcription factor A-4b-like | 6.84363 | 0.00943 | ↑ |
| A0A1S4CHI4 | protein RRP6-like 2 | 6.41399 | 0.00301 | ↑ |
| A0A1S4BZ64 | extracellular ribonuclease LE-like | 6.32291 | 0.03170 | ↑ |
| A0A1S4APB0 | phospholipase A2-alpha-like | 6.06945 | 0.04319 | ↑ |
| A0A1S3Z5Y0 | Mitogen-activated protein kinase | 5.96688 | 0.03056 | ↑ |
| A0A1S3YVW1 | heat stress transcription factor A-5-like | 5.92661 | 0.00132 | ↑ |
| A0A1S3XFX0 | Non-specific serine/threonine protein kinase | 5.26714 | 0.04176 | ↑ |
| A0A1S3ZR02 | protein SUPPRESSOR OF K(+) TRANSPORT GROWTH DEFECT 1-like | 5.16519 | 0.02990 | ↑ |
| A0A1S3Y747 | protein NUCLEAR FUSION DEFECTIVE 2-like | 5.08897 | 0.03168 | ↑ |
| A0A1S4A2M4 | GDSL esterase/lipase At5g33370-like | 5.07278 | 0.03768 | ↑ |
| A0A1S3YES2 | AP-2 complex subunit alpha | 5.04043 | 0.02425 | ↑ |
| A0A1S4BI60 | Cytochrome c oxidase subunit | 4.99680 | 0.01717 | ↑ |
| F1T161 | Berberine bridge enzyme-like protein | 4.89170 | 0.04681 | ↑ |
| A0A1S4AQV9 | probable calcium-binding protein CML14 | 4.83885 | 0.04602 | ↑ |
| A0A1S3XKI8 | uncharacterized protein LOC107766129 | 4.70264 | 0.01655 | ↑ |
| A0A1S3X2H9 | pre-mRNA-splicing factor 18-like | 4.46421 | 0.00467 | ↑ |
| A0A1S3Z3N3 | PLASMODESMATA CALLOSE-BINDING PROTEIN 5-like | 4.36462 | 0.00408 | ↑ |
| A0A1S3X808 | splicing factor U2AF-associated protein 2-like | 4.16770 | 0.00400 | ↑ |
| A0A1S4ANQ7 | mitochondrial import inner membrane translocase subunit Tim13-like | 4.09889 | 0.02617 | ↑ |
| A0A1S3ZFM5 | 60S ribosomal protein L6-like | 3.94715 | 0.04418 | ↑ |
| A0A1S3YDI6 | Peptidylprolyl isomerase | 3.86922 | 0.00063 | ↑ |
| A0A140G1S8 | Cytochrome f | 3.84401 | 0.01006 | ↑ |
| A0A1S4DMH9 | defensin J1-2-like | 3.80063 | 0.00753 | ↑ |
| A0A1S3YPE8 | AP complex subunit sigma | 3.70329 | 0.01173 | ↑ |
| A0A1S3X4E5 | Replication factor C subunit 1 | 3.69860 | 0.00462 | ↑ |
| A0A1S4AAK7 | double-stranded RNA-binding protein 4-like isoform X2 | 3.65994 | 0.00183 | ↑ |
| A0A1S3YTS8 | mitochondrial import receptor subunit TOM6 homolog | 3.64356 | 0.04061 | ↑ |
| A0A1S3X523 | rapid alkalinization factor-like | 3.63808 | 0.03797 | ↑ |
| A0A1S4DET1 | probable protein kinase At2g41970 | 3.63227 | 0.01465 | ↑ |
| A0A1S3X395 | probable plastid-lipid-associated protein 13 | 3.62110 | 0.00518 | ↑ |
| A0A1S4CF12 | epidermal growth factor receptor substrate 15-like 1 | 3.60831 | 0.04462 | ↑ |
| A0A1S3XBS6 | uncharacterized protein LOC107763315 | 3.56066 | 0.00180 | ↑ |
| A0A1S4BG77 | SEC1 family transport protein SLY1-like isoform X1 | 3.54153 | 0.02574 | ↑ |
| A0A1S4AXQ5 | pathogenesis-related protein PR-4A | 3.49504 | 0.04607 | ↑ |
| A0A1S4D7C4 | fibronectin-binding protein A-like | 3.44547 | 0.01436 | ↑ |
| A0A1S4B0C4 | probable protein Pop3 | 3.43005 | 0.04914 | ↑ |
| A0A0C4FST6 | DEF1 protein | 3.41338 | 0.02150 | ↑ |
| A0A1S3XB30 | Ribosomal protein L15 | 3.39380 | 0.00644 | ↑ |
| A0A1S3ZN92 | DNA damage-inducible protein 1 | 3.37533 | 0.04442 | ↑ |
| A0A1S3YH85 | methylcrotonoyl-CoA carboxylase beta chain, mitochondrial-like | 3.36460 | 0.03538 | ↑ |
| A0A1S4CBZ3 | protein STRUBBELIG-RECEPTOR FAMILY 3-like | 3.36188 | 0.00261 | ↑ |
| A0A1S3YFI6 | Coatomer subunit alpha | 3.34103 | 0.03516 | ↑ |
| A0A0F7R4E4 | Methyltransferase | 3.32601 | 0.01015 | ↑ |
| A0A1S3YJ34 | probable mediator of RNA polymerase II transcription subunit 26c | 3.31334 | 0.00467 | ↑ |
| Q9SP09 | DnaJ-like protein | 3.26659 | 0.04440 | ↑ |
| A0A1S3YLI1 | prosaposin-like | 3.22247 | 0.02041 | ↑ |
| H9CCH6 | Biotin carboxylase | 3.18179 | 0.03822 | ↑ |
| A0A1S4CAX0 | legumin B-like | 3.15270 | 0.02624 | ↑ |
| A0A1S3YKV1 | wound-induced basic protein | 3.14688 | 0.03812 | ↑ |
| A0A1S4CU31 | nifU-like protein 4, mitochondrial | 3.13437 | 0.04735 | ↑ |
| A0A1S4AFB0 | splicing factor 3A subunit 3-like | 3.08692 | 0.02982 | ↑ |
| A0A1S4BC16 | fasciclin-like arabinogalactan protein 1 | 3.04572 | 0.03606 | ↑ |
| A0A1S4BUY0 | Phytochrome | 2.99036 | 0.00541 | ↑ |
| A0A1S4AYE7 | UPF0426 protein At1g28150, chloroplastic | 2.93873 | 0.02404 | ↑ |
| A0A1S3XLJ2 | probable receptor-like protein kinase At5g47070 | 2.84086 | 0.03187 | ↑ |
| A0A1S4CCY5 | uncharacterized protein LOC107817723 isoform X1 | 2.83905 | 0.00347 | ↑ |
| A0A1S4AA04 | ABC transporter F family member 1-like | 2.78402 | 0.00541 | ↑ |
| A0A1S3XH44 | Clathrin light chain | 2.78277 | 0.00626 | ↑ |
| A0A1S4B8P0 | Non-specific lipid-transfer protein | 2.74409 | 0.04666 | ↑ |
| A0A1S3XMC0 | pantothenate kinase 2 | 2.74167 | 0.04749 | ↑ |
| A0A1S4CMZ0 | mannosylglycoprotein endo-beta-mannosidase-like isoform X1 | 2.73664 | 0.03556 | ↑ |
| A0A1S4A2J0 | cysteine desulfurase 1, chloroplastic-like | 2.73361 | 0.02427 | ↑ |
| A0A1S3XLD3 | calumenin-A-like | 2.72782 | 0.02509 | ↑ |
| A0A1S4CN79 | Glycosyltransferase | 2.70875 | 0.00819 | ↑ |
| A0A1S3XGA2 | beta-xylosidase/alpha-L-arabinofuranosidase 2-like | 2.70334 | 0.01760 | ↑ |
| A0A1S3X2T9 | 60S ribosomal protein L30-like | 2.66416 | 0.00010 | ↑ |
| A0A1S3XQS5 | nuclear pore complex protein NUP98A-like | 2.65538 | 0.03982 | ↑ |
| A0A1S4BH40 | uncharacterized protein LOC107808191 | 2.64233 | 0.04211 | ↑ |
| A0A1S3Z0Z2 | uncharacterized protein LOC107781840 isoform X2 | 2.61818 | 0.00301 | ↑ |
| A0A1S3ZB60 | putative ripening-related protein 2 | 2.59566 | 0.01724 | ↑ |
| A0A1S4DNT1 | Eukaryotic translation initiation factor 3 subunit I | 2.57305 | 0.01327 | ↑ |
| A0A1S3X0M4 | Purple acid phosphatase | 2.55775 | 0.00734 | ↑ |
| A0A1S3XJ54 | NADH dehydrogenase [ubiquinone] 1 beta subcomplex subunit 10-A-like | 2.54768 | 0.03262 | ↑ |
| A0A1S3XLU0 | mitochondrial import inner membrane translocase subunit TIM44-2-like | 2.53635 | 0.02924 | ↑ |
| A0A1S4C5X4 | oxygen-dependent coproporphyrinogen-III oxidase, chloroplastic | 2.49230 | 0.03872 | ↑ |
| A0A1S4BCE1 | uncharacterized protein LOC107806770 | 2.48221 | 0.00166 | ↑ |
| A0A1S4APS2 | protein phosphatase 1 regulatory subunit pprA-like | 2.47188 | 0.03280 | ↑ |
| A0A1S4CFC4 | ACT domain-containing protein ACR12-like | 2.46935 | 0.01290 | ↑ |
| A0A1S3YP25 | protein BTR1-like isoform X3 | 2.45821 | 0.01838 | ↑ |
| A0A0S0N4U0 | Tubulin alpha chain | 2.42074 | 0.00270 | ↑ |
| A0A1S3X5T5 | ethylene-insensitive protein 2-like | 2.40460 | 0.01943 | ↑ |
| A0A1S3YPS6 | GDSL esterase/lipase At1g29670-like | 2.40286 | 0.04627 | ↑ |
| A0A1S3X2I0 | thioredoxin Y1, chloroplastic-like | 2.38865 | 0.01397 | ↑ |
| A0A1S4BST6 | vesicle-associated membrane protein 722-like | 2.38722 | 0.00511 | ↑ |
| A0A1S3YSG1 | uncharacterized protein LOC107779229 isoform X2 | 2.38123 | 0.04821 | ↑ |
| A0A1S4CBD4 | probable LRR receptor-like serine/threonine-protein kinase At1g06840 isoform X2 | 2.38094 | 0.01028 | ↑ |
| A0A1S3X5Z8 | uncharacterized protein LOC107761618 | 2.38003 | 0.01245 | ↑ |
| A0A1S4B4G8 | isocitrate dehydrogenase [NAD] regulatory subunit 1, mitochondrial-like | 2.37686 | 0.03681 | ↑ |
| A0A1S3XV64 | Vacuolar protein sorting-associated protein 28 homolog | 2.37231 | 0.01204 | ↑ |
| A0A1S3YH03 | ABSCISIC ACID-INSENSITIVE 5-like protein 7 | 2.35855 | 0.02955 | ↑ |
| A0A1S3YLS5 | peroxisomal (S)-2-hydroxy-acid oxidase-like isoform X1 | 2.35002 | 0.00291 | ↑ |
| A0A1S4C432 | Structural maintenance of chromosomes protein | 2.34612 | 0.01411 | ↑ |
| A0A1S3XPL6 | bifunctional riboflavin kinase/FMN phosphatase-like | 2.32390 | 0.00155 | ↑ |
| A0A1S3XGK9 | 1-aminocyclopropane-1-carboxylate oxidase 1-like | 2.32037 | 0.00397 | ↑ |
| A0A1S3Z1S8 | probable fructokinase-7 | 2.31576 | 0.00211 | ↑ |
| A0A1S3XA26 | cysteine desulfurase, mitochondrial-like | 2.29552 | 0.02999 | ↑ |
| A0A097BTZ1 | ATP-dependent Clp protease proteolytic subunit | 2.28634 | 0.01537 | ↑ |
| A0A1S3YCL4 | putative protease Do-like 14 | 2.27497 | 0.00857 | ↑ |
| A0A1S4CK96 | miraculin-like | 2.26866 | 0.03720 | ↑ |
| A0A1S4AP10 | probable Xaa-Pro aminopeptidase 3 | 2.26829 | 0.01874 | ↑ |
| A0A1S4BI16 | defensin Lc-def-like | 2.26568 | 0.02561 | ↑ |
| A0A1S3Y6I9 | defensin J1-2-like | 2.25492 | 0.04436 | ↑ |
| A0A1S4AEU7 | uncharacterized protein LOC107796902 | 2.24813 | 0.04514 | ↑ |
| A0A1S4BNR1 | nuclear pore complex protein NUP62-like | 2.22843 | 0.00763 | ↑ |
| A0A1S4A9D6 | outer envelope protein 80, chloroplastic-like isoform X2 | 2.20466 | 0.00164 | ↑ |
| A0A1S3XUF6 | Mitochondrial fission 1 protein | 2.19614 | 0.00839 | ↑ |
| A0A1S4B6N0 | L-gulonolactone oxidase 3-like | 2.19335 | 0.04391 | ↑ |
| A0A1S3XAF1 | ISWI chromatin-remodeling complex ATPase CHR11-like | 2.19329 | 0.00565 | ↑ |
| A0A1S4D3U3 | aspartic proteinase-like protein 2 isoform X2 | 2.18105 | 0.04953 | ↑ |
| A0A1S3ZYJ3 | Uricase | 2.16981 | 0.02470 | ↑ |
| A0A1S4AC68 | glutathione S-transferase T1-like | 2.16469 | 0.00666 | ↑ |
| A0A1S3XCD1 | V-type proton ATPase subunit | 2.15981 | 0.04409 | ↑ |
| A0A1S4BDV2 | clustered mitochondria protein-like | 2.13847 | 0.02221 | ↑ |
| A0A1S3Z835 | allene oxide synthase, chloroplastic-like | 2.13792 | 0.03984 | ↑ |
| A0A1S4DEU5 | cyprosin-like | 2.13554 | 0.01407 | ↑ |
| A0A0K0XR60 | Autophagy-related protein 3 | 2.13185 | 0.01401 | ↑ |
| A0A1S4BB77 | GTP-binding protein SAR2 | 2.12032 | 0.03401 | ↑ |
| A0A1S4DLU6 | subtilisin-like protease SBT1.6 | 2.09838 | 0.02020 | ↑ |
| A0A1S4B0A4 | uncharacterized protein LOC107803234 isoform X1 | 2.08211 | 0.04380 | ↑ |
| A0A1S3X239 | 18.8 kDa class II heat shock protein-like | 2.07002 | 0.04308 | ↑ |
| A0A1S3Y2Y7 | cystathionine gamma-synthase 1, chloroplastic-like | 2.06762 | 0.01528 | ↑ |
| A0A1S3YAQ4 | nuclear transport factor 2-like | 2.06447 | 0.02775 | ↑ |
| A0A1S3Z3L4 | exocyst complex component SEC3A-like | 2.05504 | 0.04470 | ↑ |
| A0A1S3ZG71 | TBC1 domain family member 13-like | 2.03254 | 0.02629 | ↑ |
| A0A1S3XRX5 | D-amino-acid transaminase, chloroplastic-like isoform X1 | 2.03070 | 0.00622 | ↑ |
| A0A1S3ZC53 | malonate--CoA ligase-like | 2.02755 | 0.00627 | ↑ |
| A0A1S3Y8V8 | glutathione S-transferase DHAR3, chloroplastic | 2.00906 | 0.02644 | ↑ |
| A0A1S4DA59 | V-type proton ATPase subunit E-like | 1.98189 | 0.02599 | ↑ |
| A0A0A8WH00 | Aquaporin PIP1 5 | 1.96548 | 0.00571 | ↑ |
| A0A1S3YGR9 | Malate dehydrogenase | 1.95577 | 0.00565 | ↑ |
| A0A1S4C7B8 | ubiquitin-conjugating enzyme E2 27-like | 1.95524 | 0.02648 | ↑ |
| A0A1S3Z0F7 | putative transferase At4g12130, mitochondrial isoform X1 | 1.93485 | 0.03930 | ↑ |
| A0A1S3XTU2 | Peroxidase | 1.92299 | 0.03734 | ↑ |
| A0A1S4AFV7 | 4-coumarate--CoA ligase 2 | 1.92220 | 0.02614 | ↑ |
| A0A1S4CB73 | quinolinate synthase, chloroplastic-like | 1.92029 | 0.02191 | ↑ |
| A0A1S4A3B3 | Delta-aminolevulinic acid dehydratase | 1.90561 | 0.00374 | ↑ |
| A0A1S4D3X7 | alpha-L-fucosidase 1-like | 1.88059 | 0.00289 | ↑ |
| A0A1S3XA42 | acyl-coenzyme A oxidase 4, peroxisomal isoform X1 | 1.88020 | 0.03927 | ↑ |
| A0A1S4BJ43 | probable 3-hydroxyisobutyrate dehydrogenase-like 1, mitochondrial | 1.86747 | 0.04446 | ↑ |
| A0A1S3XZ22 | ATP-dependent Clp protease proteolytic subunit | 1.84327 | 0.01733 | ↑ |
| A0A1S3Y9C6 | uncharacterized protein LOC107773869 isoform X1 | 1.83354 | 0.00193 | ↑ |
| A0A1S4BTR8 | dynamin-related protein 1E-like | 1.82856 | 0.03339 | ↑ |
| A0A1S3XL06 | Amidophosphoribosyltransferase | 1.81186 | 0.01985 | ↑ |
| A0A1S4A439 | ABC transporter E family member 2-like | 1.73332 | 0.03672 | ↑ |
| A0A1S4BBS1 | nucleosome assembly protein 14 isoform X3 | 1.73126 | 0.02507 | ↑ |
| High-parent Expression | | | |  |
| A0A1S3Y3V1 | 2-oxoglutarate-dependent dioxygenase DAO-like | 2.04084 | 0.01432 | ↑ |
| A0A1S3WYU5 | B-cell receptor-associated protein 31-like | 1.99222 | 0.00676 | ↑ |
| A0A1S4AQR9 | subtilisin-like protease SBT1.7 | 1.98099 | 0.02658 | ↑ |
| A0A1S3ZF94 | ATP-dependent zinc metalloprotease FTSH 10, mitochondrial-like | 1.98009 | 0.02649 | ↑ |
| A0A1S3Y274 | protein SLOW GREEN 1, chloroplastic-like | 1.97907 | 0.01732 | ↑ |
| A0A1S3XHR3 | uncharacterized protein LOC107765337 | 1.92923 | 0.03928 | ↑ |
| A0A1S3XDP9 | peroxiredoxin-2B-like | 1.92667 | 0.01375 | ↑ |
| A0A1S3X155 | WPP domain-interacting tail-anchored protein 1-like isoform X2 | 1.92140 | 0.01996 | ↑ |
| O24162 | 5-epi-aristolochene synthase (Fragment) | 1.90474 | 0.04542 | ↑ |
| A0A1S4BLL1 | alpha-L-arabinofuranosidase 1-like | 1.90093 | 0.02507 | ↑ |
| A0A1S3WYJ4 | NADH dehydrogenase [ubiquinone] 1 beta subcomplex subunit 9-like | 1.88867 | 0.03304 | ↑ |
| A0A1S3YGW5 | solanesyl diphosphate synthase 3, chloroplastic/mitochondrial-like isoform X2 | 1.83064 | 0.02462 | ↑ |
| A0A1S4A824 | ATP-dependent Clp protease proteolytic subunit | 1.81133 | 0.01841 | ↑ |
| A0A1S4DGN5 | Superoxide dismutase | 1.81031 | 0.03834 | ↑ |
| A0A1S3ZQX9 | prolyl endopeptidase-like | 1.78859 | 0.01176 | ↑ |
| A0A1S4D5N8 | anthranilate phosphoribosyltransferase isoform X1 | 1.78440 | 0.04330 | ↑ |
| A0A1S4BBF2 | thioredoxin domain-containing protein 9 homolog | 1.76071 | 0.04511 | ↑ |
| A0A1S4AJU5 | uncharacterized protein LOC107798473 isoform X2 | 1.75634 | 0.01881 | ↑ |
| A0A1S4CES4 | Threonine dehydratase | 1.74939 | 0.01594 | ↑ |
| A0A1S4A9C8 | apoptosis inhibitor 5-like isoform X1 | 1.72505 | 0.00947 | ↑ |
| A0A1S3XW26 | (+)-neomenthol dehydrogenase-like | 1.72192 | 0.03352 | ↑ |
| A0A1S3Y2K8 | beta-ureidopropionase-like | 1.71983 | 0.03698 | ↑ |
| A0A1S3YUL3 | probable glutathione S-transferase | 1.71917 | 0.04236 | ↑ |
| A0A1S3ZJS9 | Pyruvate kinase | 1.71786 | 0.03743 | ↑ |
| A0A1S3XKM0 | Glutamine synthetase | 1.70305 | 0.02842 | ↑ |
| A0A1S3X6W9 | Pyrophosphate--fructose 6-phosphate 1-phosphotransferase subunit alpha | 1.70287 | 0.01131 | ↑ |
| A0A1S3ZH17 | basic 7S globulin-like | 1.65919 | 0.01092 | ↑ |
| A0A1S3Z5P8 | mitochondrial acidic protein MAM33-like isoform X1 | 1.65344 | 0.04140 | ↑ |
| A0A1S4A8H3 | protein EMSY-LIKE 3-like isoform X2 | 1.65331 | 0.02192 | ↑ |
| A0A1S4DFP5 | 26S proteasome non-ATPase regulatory subunit 12 homolog A-like | 1.65045 | 0.00695 | ↑ |
| A0A1S4A1V3 | Succinate-semialdehyde dehydrogenase | 1.64177 | 0.03481 | ↑ |
| A0A1S4DB13 | probable fructokinase-6, chloroplastic | 1.64030 | 0.00685 | ↑ |
| A0A1S3ZMG1 | Serine/threonine-protein phosphatase 2A 55 kDa regulatory subunit B | 1.63473 | 0.02284 | ↑ |
| A0A1S3X7V8 | probable 2,3-bisphosphoglycerate-independent phosphoglycerate mutase | 1.61510 | 0.04988 | ↑ |
| A0A1S3YKI8 | Succinate dehydrogenase [ubiquinone] flavoprotein subunit, mitochondrial | 1.59232 | 0.02831 | ↑ |
| A0A1S3XGH3 | Amine oxidase | 1.59016 | 0.04806 | ↑ |
| A0A1S4BH12 | 40S ribosomal protein S27 | 1.58262 | 0.02915 | ↑ |
| A0A1S3XVA1 | splicing factor 3B subunit 1-like | 1.58168 | 0.00733 | ↑ |
| A0A1S4CU78 | Serine/threonine-protein phosphatase | 1.58094 | 0.01327 | ↑ |
| A0A1S3X611 | probable UDP-arabinopyranose mutase 5 | 1.57686 | 0.00299 | ↑ |
| A0A1S3ZFG7 | probable 2-oxoglutarate-dependent dioxygenase AOP1 | 1.57061 | 0.00796 | ↑ |
| A0A1S4AJF4 | Ubiquitin carboxyl-terminal hydrolase | 1.56743 | 0.03087 | ↑ |
| A0A1S4CCB8 | vesicle-fusing ATPase-like | 1.55787 | 0.02039 | ↑ |
| A0A1S3Z362 | acylamino-acid-releasing enzyme-like isoform X2 | 1.54893 | 0.00670 | ↑ |
| A0A1S4CPD1 | Uridine kinase | 1.54369 | 0.03359 | ↑ |
| A0A1S3YYG2 | Glycine cleavage system P protein | 1.53202 | 0.01363 | ↑ |
| A0A1S4BBK3 | glutelin type-B 2-like | 1.53101 | 0.00971 | ↑ |
| A0A1S3YMX2 | uncharacterized protein LOC107777802 | 1.53007 | 0.03788 | ↑ |
| A0A1S3YUZ1 | Pyrophosphate--fructose 6-phosphate 1-phosphotransferase subunit beta | 1.52680 | 0.01656 | ↑ |
| A0A1S3WYM6 | actin-101 | 1.52591 | 0.02652 | ↑ |
| A0A1S3ZR64 | probable cinnamyl alcohol dehydrogenase 1 | 1.52412 | 0.02781 | ↑ |
| Low-parent Expression | | | |  |
| A0A1S4BT83 | KH domain-containing protein At4g18375-like | 0.66244 | 0.03746 | ↓ |
| A0A1S3YTZ2 | Ketol-acid reductoisomerase | 0.65809 | 0.03644 | ↓ |
| A0A1S4BRZ6 | endonuclease 4-like | 0.65177 | 0.03690 | ↓ |
| A0A1S3XDM4 | calumenin-like | 0.64946 | 0.04455 | ↓ |
| A0A1S4DKZ0 | uncharacterized protein LOC107830885 isoform X2 | 0.64858 | 0.03192 | ↓ |
| A0A1S3ZZL0 | enolase-like | 0.64233 | 0.02514 | ↓ |
| A0A1S3Z550 | transcription factor Pur-alpha 1-like isoform X2 | 0.63800 | 0.01174 | ↓ |
| E2F3S8 | G-strand specific single-stranded telomere-binding protein 1 | 0.62811 | 0.04682 | ↓ |
| A0A1S3X3D2 | lanC-like protein GCL1 | 0.61899 | 0.02475 | ↓ |
| A0A068JFR6 | Triosephosphate isomerase | 0.61711 | 0.03812 | ↓ |
| A0A1S4A4G2 | RNA-binding protein 8A-like | 0.60660 | 0.04898 | ↓ |
| A0A1S3XYS0 | NAP1-related protein 2-like | 0.60428 | 0.04343 | ↓ |
| A0A1S3Z6E6 | V-type proton ATPase subunit H | 0.59291 | 0.01246 | ↓ |
| A0A1S3YJJ6 | glucan endo-1,3-beta-glucosidase 9-like | 0.58801 | 0.01016 | ↓ |
| A0A1S4D8A6 | Protein disulfide-isomerase | 0.58383 | 0.02162 | ↓ |
| A0A1S4DNP9 | Peroxidase | 0.57991 | 0.02452 | ↓ |
| A0A1S4BUH8 | lysM domain receptor-like kinase 4 | 0.57769 | 0.03178 | ↓ |
| A0A1S4AC92 | UBP1-associated protein 2B-like | 0.57560 | 0.04411 | ↓ |
| A0A1S3YNY9 | dynamin-related protein 1E-like | 0.57409 | 0.03299 | ↓ |
| A0A1S3YJQ9 | nucleolin 1-like isoform X2 | 0.56971 | 0.01227 | ↓ |
| A0A1S3XJM8 | heterogeneous nuclear ribonucleoprotein 1-like | 0.56893 | 0.04101 | ↓ |
| A0A1S3YGA9 | glutamine synthetase nodule isozyme-like | 0.54709 | 0.01176 | ↓ |
| A0A1S4B881 | luminal-binding protein | 0.54403 | 0.04947 | ↓ |
| A0A1S4BUT1 | early nodulin-like protein 1 | 0.54056 | 0.04727 | ↓ |
| Under-dominance Expression | | | |  |
| A0A1S3X313 | uncharacterized protein LOC107760706 | 0.53086 | 0.04497 | ↓ |
| A0A1S3XH24 | REF/SRPP-like protein At1g67360 | 0.52979 | 0.00550 | ↓ |
| A0A1S3ZHA4 | translin-like | 0.52598 | 0.01946 | ↓ |
| A0A1S3Y3X5 | SURF1-like protein | 0.52192 | 0.00073 | ↓ |
| A0A1S3YUH0 | 60S ribosomal protein L4-1-like | 0.51831 | 0.01671 | ↓ |
| A0A1S4D1U0 | 60S ribosomal protein L18a | 0.51458 | 0.03885 | ↓ |
| A0A1S4DND2 | Protein disulfide-isomerase | 0.51022 | 0.01876 | ↓ |
| A0A1S4B0R9 | Trafficking protein particle complex subunit | 0.49185 | 0.01284 | ↓ |
| A0A1S3Y0R2 | histone chaperone ASF1B-like | 0.47846 | 0.01297 | ↓ |
| A0A1S3Y1L6 | cytochrome b561 and DOMON domain-containing protein At3g25290-like | 0.47721 | 0.02280 | ↓ |
| A0A1S4DD92 | glucan endo-1,3-beta-glucosidase 8-like | 0.47214 | 0.01707 | ↓ |
| A0A1S3ZC38 | Proteasome subunit beta | 0.46729 | 0.01597 | ↓ |
| A0A1S4DJJ7 | Peroxidase | 0.46713 | 0.00617 | ↓ |
| A0A1S3X241 | phospholipase A-2-activating protein-like | 0.46461 | 0.00802 | ↓ |
| A0A1S4CSA0 | PI-PLC X domain-containing protein At5g67130-like | 0.45966 | 0.02686 | ↓ |
| A0A1S3XV28 | extracellular ribonuclease LE-like isoform X1 | 0.45885 | 0.04616 | ↓ |
| A0A1S4DD20 | aminoacylase-1-like | 0.45822 | 0.04916 | ↓ |
| A0A1S3Z9T6 | Peroxidase | 0.45633 | 0.01291 | ↓ |
| A0A1S4CT85 | binding partner of ACD11 1-like | 0.44937 | 0.04843 | ↓ |
| A0A1S3X3B5 | protein TPLATE | 0.44669 | 0.02598 | ↓ |
| A0A1S3Y048 | Peroxidase | 0.43910 | 0.03249 | ↓ |
| A0A1S3XQB7 | Peroxidase | 0.43697 | 0.00450 | ↓ |
| A0A1S4APB4 | Alpha-galactosidase | 0.43090 | 0.03172 | ↓ |
| Q50LG5 | Peroxidase | 0.41185 | 0.00643 | ↓ |
| A0A1S4A594 | Peroxidase | 0.40910 | 0.03695 | ↓ |
| A0A1S3ZDD8 | histone H1-like isoform X1 | 0.40678 | 0.03967 | ↓ |
| A0A1S3Y5P8 | serpin-ZX-like | 0.40295 | 0.04601 | ↓ |
| A0A1S4DQ97 | glycine-rich protein 2-like | 0.39839 | 0.00525 | ↓ |
| A0A1S3Y7P7 | polyphenol oxidase E, chloroplastic-like | 0.39826 | 0.03598 | ↓ |
| A0A1S4A107 | Peroxidase | 0.39774 | 0.04322 | ↓ |
| A0A1S3YEI2 | syntaxin-132-like | 0.39535 | 0.01752 | ↓ |
| A0A076L2G1 | Sulfurtransferase | 0.39189 | 0.00144 | ↓ |
| A0A1S3Y8J6 | Phosphomannomutase | 0.39019 | 0.01456 | ↓ |
| A0A1S3ZDR0 | Peroxidase | 0.35936 | 0.04772 | ↓ |
| A0A1S4CYK3 | FAM10 family protein At4g22670-like | 0.35362 | 0.01220 | ↓ |
| A0A1S4AXG2 | GrpE protein homolog | 0.35160 | 0.00934 | ↓ |
| A0A1S4DGG9 | mavicyanin-like | 0.34658 | 0.03424 | ↓ |
| A0A1S4AWR5 | benzaldehyde dehydrogenase (NAD(+))-like | 0.34446 | 0.00478 | ↓ |
| A0A1S4A171 | fasciclin-like arabinogalactan protein 7 | 0.33850 | 0.04379 | ↓ |
| A0A1S3Y3V3 | wall-associated receptor kinase-like 20 | 0.33657 | 0.00880 | ↓ |
| A0A1S4DEF0 | Peroxidase | 0.31955 | 0.01344 | ↓ |
| Q94IQ1 | Peroxidase | 0.28072 | 0.03062 | ↓ |
| A0A1S3X813 | Germin-like protein | 0.24709 | 0.03236 | ↓ |
| A0A1S3YGY8 | protein RCC2 homolog | 0.22591 | 0.02342 | ↓ |
| A0A1S4BHI8 | succinate dehydrogenase subunit 5, mitochondrial-like | 0.21118 | 0.01020 | ↓ |

Supplementary table 2. The identified additive and non-additive accumulation proteins in hybrids. The P value of the significance test was derived from the T two-tailed test. P＜0.05 represents significant difference, and P＜0.01 represents extremely significant difference. The foldchange indicated the multiple of the expression difference of the hybrids compared to the mid-parent value. The foldchange value greater than 1.5 was defined as an up-regulated expression, and less than 0.67 was defined as a down-regulated expression.

| Accession | Encoded protein | Primer sequence | Reverse primer |
| --- | --- | --- | --- |
| gene_24753 | Actin-1 | GTGTTATGGTCGGAATGGG | TGGAACGCTAAATGTCTCAAA |
| A0A1S4BJT3 | AO | ACTGGTGATGGAATGGCTATGG | TCTCTGGCACTTGGTATGTTGG |
| A0A1S4CB73 | QS | GGATGTGCCTCCTGTCCATAT | GAATCGTCCTGCCTCATAAGC |
| F1T161 | BBL | CCTACGCTTGGTTCAACGC | GACACAACTTCCACCCTTGTG |
| A0A1S4AA04 | ABCF1 | CTGGATTGGGCGAGTGAATC | CAACGACCAAGAGGAAATAATGAA |
| A0A1S4BG77 | SLY1 | AGATGGTGGCATCCCTGTTG | CTTGTCTCCTGGCACGCTTA |

Supplementary Table 3. Primer sequences of genes used in real-time PCR analysis. Sequences designed using primer 5 software.


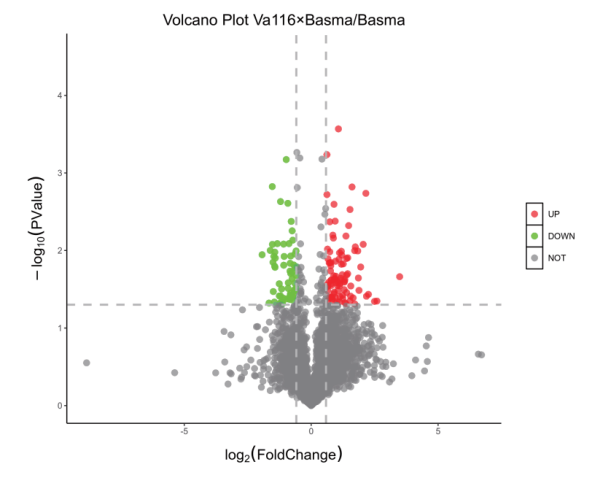


b


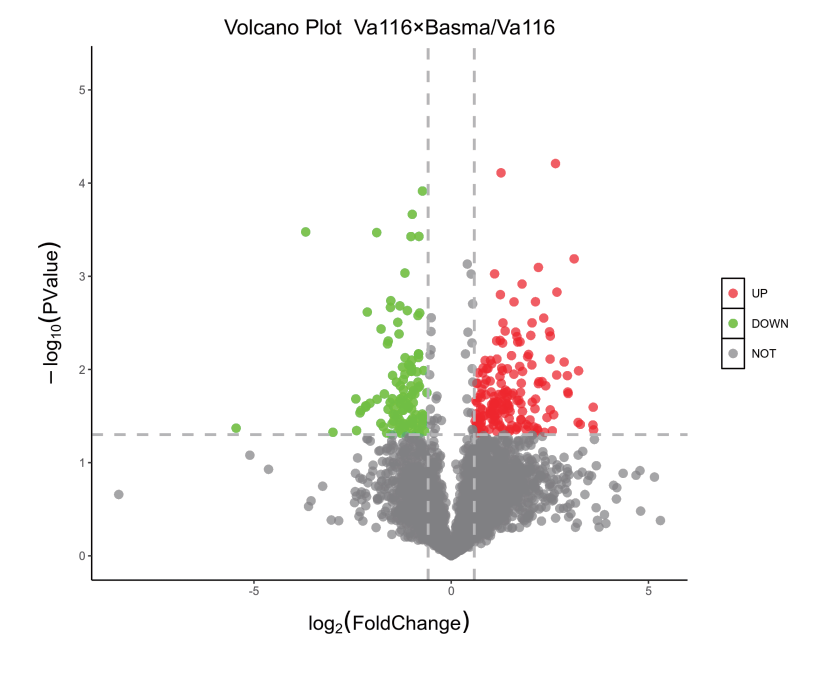


a

Supplementary figure 1. The Volcano of differentially expressed proteins. a: Comparison between hybrid va116 × Basma and female parent Va116. b: Comparison between hybrid va116 × Basma and male parent va116. The horizontal ordinate was the difference multiple (logarithmic transformation based on 2), the Y-axis was the significance p-value of the difference (logarithmic transformation based on 10), the red dot in the figure was the protein with significant up-regulation expression (multiple change was greater than 1.2 times and P value < 0.05), the green dot in the figure was the protein with significant down-regulation expression (multiple change was lower than 0.67 times and P value < 0.05), and the gray dot is the protein without difference change. Figure generated using RStudio software loaded with the ggplot package.

Attachment 1. Quantitative list of protein identification. Accessions indicated the protein number in the protein sequence database ( FASTA database ). Descriptions was a protein function description in a protein sequence-based database.

Attachment 2. GO function annotation statistics table. GO_ID: the ID of the annotated GO term. Term: the name of the annotated GO term. Category: the category of the annotated GO term ( P: Biology Process; F: Molecular Function; C: Cellular Component ). Test: the number of proteins associated with this GO term in the target protein set. Ref: the number of proteins associated with this GO term in the background protein set. TestAll: the total number of proteins in the target protein set. RefAll: the total number of proteins in the background protein set. Test_per: The proportion of proteins associated with this GO term in the target protein set. Ref_per: The proportion of proteins associated with this GO term in the background protein set. Over_Under: the proportion of target proteins associated with this GO term was relatively high relative to the proportion of background proteins associated with this GO term (Test_per > Ref_per) or low (Test_per < Ref_per), usually retained only Over-represented GO term in the target protein set. TestSeqs: the protein ID associated with this GO term in the target protein set. RefSeqs: the protein ID associated with this GO term in the background protein set. P value: the significance index of enrichment analysis, the smaller p-value indicateed that the GO term was more significantly affected under specific biological treatment, which was calculated by hypergeometric distribution. FDR: false discovery rate, error control index in multiple hypothesis test, was a Benjamin Hochberg correction for p-value. RichFactor: the ratio of the number of proteins associated with a pathway in the target protein set to the number of proteins associated with that pathway in the background protein set.

Attachment 3. KEGG channel annotation statistics table. Map_ID: the pathway ID in which the target protein might be involved. Map_Name: the name of the pathway in which the target protein might participated. Test: the number of proteins associated with this GO term in the target protein set. Ref: the number of proteins associated with this GO term in the background protein set. TestAll: the total number of proteins in the target protein set. RefAll: the total number of proteins in the background protein set. Test_per: The proportion of proteins associated with this GO term in the target protein set. Ref_per: The proportion of proteins associated with this GO term in the background protein set. Over_Under: the proportion of target proteins associated with this GO term was relatively high relative to the proportion of background proteins associated with this GO term (Test_per > Ref_per) or low (Test_per < Ref_per), usually retained only Over-represented GO term in the target protein set . TestSeqs: the protein ID associated with this GO term in the target protein set. RefSeqs: the protein ID associated with this GO term in the background protein set. P value: the significance index of enrichment analysis, the smaller p-value indicateed that the GO term was more significantly affected under specific biological treatment, which was calculated by hypergeometric distribution. FDR: false discovery rate, error control index in multiple hypothesis test, was a Benjamin Hochberg correction for p-value. RichFactor: the ratio of the number of proteins associated with a pathway in the target protein set to the number of proteins associated with that pathway in the background protein set.
